# Characterizing the Effects of Glutathione as an Immunoadjuvant in the Treatment of Tuberculosis

**DOI:** 10.1128/AAC.01132-18

**Published:** 2018-10-24

**Authors:** Ruoqiong Cao, Garrett Teskey, Hicret Islamoglu, Rachel Abrahem, Shalok Munjal, Karo Gyurjian, Li Zhong, Vishwanath Venketaraman

**Affiliations:** a College of Life Sciences, Hebei University, Baoding, Hebei, China; b Department of Basic Medical Sciences, College of Osteopathic Medicine of the Pacific, Western University of Health Sciences, Pomona, California, USA; c Graduate College of Biomedical Sciences, Western University of Health Sciences, Pomona, California, USA; d Department of Biological Sciences, California State Polytechnic University, Pomona, California, USA

**Keywords:** Mycobacterium tuberculosis, additive effects, cytokines, glutathione, macrophages, synergistic effects, tuberculosis

## Abstract

Mycobacterium tuberculosis is the etiological agent that is responsible for causing tuberculosis (TB), which continues to affect millions of people worldwide, and the rate of resistance of M. tuberculosis to antibiotics is ever increasing. We tested the synergistic effects of *N*-acetyl cysteine (NAC; the precursor molecule for the synthesis of glutathione [GSH]) and individual first-line antibiotics typically given for the treatment of TB, such as isoniazid (INH), rifampin (RIF), ethambutol (EMB), and pyrazinamide (PZA), to improve the ability of macrophages to control intracellular M. tuberculosis infection. GSH, a pleiotropic antioxidant molecule, has previously been shown to display both antimycobacterial and immune-enhancing effects. Our results indicate that there was not only an increase in beneficial immunomodulatory effects but also a greater reduction in the intracellular viability of M. tuberculosis when macrophages were treated with the combination of antibiotics (INH, RIF, EMB, or PZA) and NAC.

## Abstract (precis)

Mycobacterium tuberculosis is the etiological agent that is responsible for causing tuberculosis (TB), which continues to affect millions of people worldwide, and the rate of resistance of M. tuberculosis to antibiotics is ever increasing. We tested the synergistic effects of *N*-acetyl cysteine (NAC; the precursor molecule for the synthesis of glutathione [GSH]) and individual first-line antibiotics typically given for the treatment of TB, such as isoniazid (INH), rifampin (RIF), ethambutol (EMB), and pyrazinamide (PZA), to improve the ability of macrophages to control intracellular M. tuberculosis infection.

## INTRODUCTION

Mycobacterium tuberculosis is the causative agent of tuberculosis (TB) and a leading cause of death worldwide (1). According to the World Health Organization (WHO), TB is currently the ninth leading cause of mortality worldwide and the principal cause of death due to a single infectious agent (2). The WHO reported that 10.4 million people contracted an active M. tuberculosis infection in 2016 alone (2). M. tuberculosis infection is acquired via inhalation of respiratory droplets, leading to the seeding of the bacteria within the lungs. More specifically, once M. tuberculosis enters the lower respiratory tract, it is engulfed by alveolar macrophages and becomes an intracellular pathogen (3). At this point, a competent immune system mounts an attack against the bacteria, either killing them off completely or, more characteristically, sequestering the bacteria within a specialized immune structure known as a granuloma, archetypally localized within the lungs (4). This process of mycobacterial containment within a granuloma is referred to as latent TB and is observed in the majority of TB cases. A granuloma consists of a multitude of immune cells, including macrophages, epithelioid histiocytes, dendritic cells, T cells, and natural killer (NK) cells, which come together to orchestrate this protective immune response and which isolate the bacteria, rendering them latent (5). The accumulation of these immune cells is mediated by various cytokines, including tumor necrosis factor alpha (TNF-α), interleukin-6 (IL-6), IL-12, IL-2, and gamma interferon (6).

However, in an immunocompromised individual, M. tuberculosis granulomas can undergo liquefaction, resulting in an active M. tuberculosis infection (7). Individuals with active TB not only are contagious but also are at a serious risk for developing permanent morbidity due to vast cellular damage. Therefore, the Centers for Disease Control and Prevention (CDC) recommends that the preferred treatment regimen for active TB be the combined administration of the antibiotics isoniazid (INH), rifampin (RIF), pyrazinamide (PZA), and ethambutol (EMB) for 2 months (the intensive phase), followed by the administration of INH and RIF for 4 months (the continuation phase) (8–10). These antibiotics are primarily used in combination to prevent the bacteria from developing resistance. However, TB treatment has been experiencing a rising risk of failure due to the development of multidrug-resistant strains of M. tuberculosis, largely due to noncompliance and inadequate adherence to standard treatment protocols and the necessary cessation of treatment due to the detrimental side effects that can be associated with the use of the aforementioned antibiotics (11–19). In search of a novel therapeutic agent to augment the treatment of TB, we investigated the synergistic effects of the glutathione (GSH) precursor *N*-acetyl cysteine (NAC) in conjunction with the individual aforementioned first-line antibiotics in promoting the macrophage-mediated killing of M. tuberculosis. NAC has been widely used for several years to enhance the intracellular levels of GSH (20). GSH, a tripeptide comprised of glutamate, cysteine, and glycine, functions to protect the cells and tissues from oxidative damage, thereby restoring redox homeostasis within the body (21). Further studies have demonstrated that the biological antioxidant GSH has both antimycobacterial effects and immune-modulating properties (22–24).

Venketaraman's laboratory has previously reported that the levels of GSH are significantly decreased in red blood cells, NK cells, macrophages, and T cells derived from the peripheral blood of individuals with HIV infection (25–27). Furthermore, we have also demonstrated that the levels of GSH are significantly compromised in brain tissue samples derived from the frontal cortex of individuals with HIV infection (22). The decreased levels of GSH among HIV-infected individuals correlated with the diminished control of M. tuberculosis infection (22–24). Additional studies have shown that the levels of GSH are significantly compromised in individuals with type 2 diabetes (T2DM) due to the diminished levels of enzymes involved in GSH synthesis (28). Findings from our clinical trials indicate that supplementation with liposomal glutathione (L-GSH) restored redox homeostasis, induced a cytokine balance, and improved immune responses against M. tuberculosis infection (24). Additionally, NAC has also been shown to have direct mycobactericidal effects (29).

Therefore, we hypothesized that treatment of THP-1 cells with NAC in conjunction with any one of the first-line antibiotics, INH, RIF, EMB, or PZA, would improve the ability of macrophages to effectively control M. tuberculosis infection. Our study findings indicate a greater reduction in the intracellular viability of M. tuberculosis when macrophages are treated with the combination of NAC and antibiotics (INH, RIF, EMB, or PZA).

## RESULTS

### Levels of total GSH in uninfected and M. tuberculosis-infected THP-1 cells.

**FIG 1:**
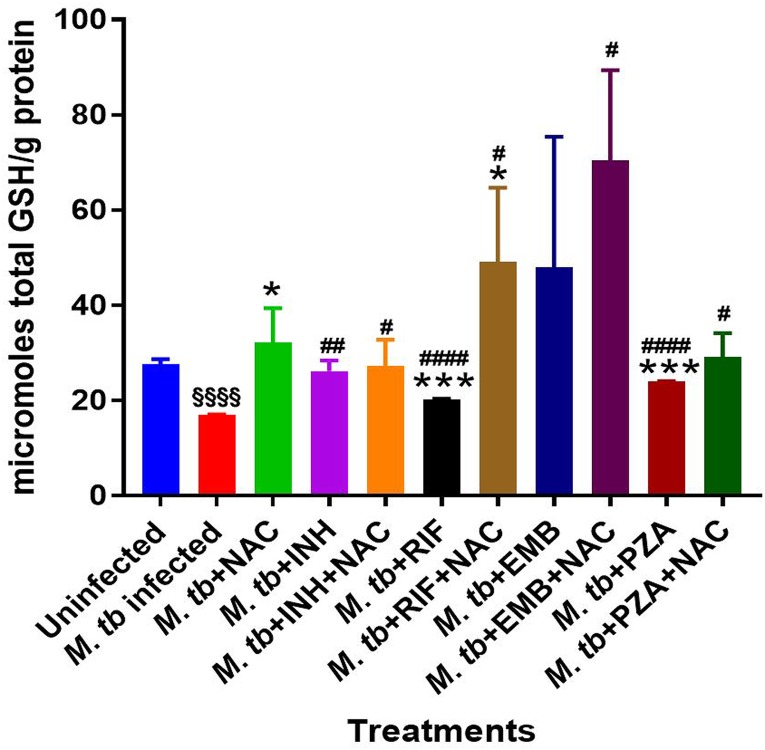
Levels of total GSH in M. tuberculosis (*M. tb*)-infected macrophages treated with first-line antibiotics in the presence and absence of NAC. The GSH assay was performed by the colorimetric method using an assay kit from Arbor Assays. Corrections were made to the total amount of protein measured by use of a bicinchoninic acid protein assay kit from Thermo Scientific. GSH and total protein levels were measured from the cellular lysates. There was a notable decrease in the levels of GSH when THP-1 cells were infected with the Erdman strain of M. tuberculosis. There was a significant increase in the levels of GSH when M. tuberculosis-infected samples were treated with NAC. A significant increase in the levels of GSH was also observed when M. tuberculosis-infected macrophages were treated with INH, INH plus NAC, RIF, RIF plus NAC, EMB plus NAC, PZA, and PZA plus NAC. Data represent the means ± SE from 6 trials. *, significant difference between the antibiotic- and NAC-treated group and the group treated with antibiotic alone and between the infected NAC-treated group and the infected control group; §, significant difference between the infected control group and the uninfected control group; #, significant difference between any group and the infected control group.

The levels of total GSH were shown to be significantly diminished in M. tuberculosis-infected macrophages compared to the levels in the uninfected control group (Fig. 1; Table 1). These results indicate that infection with the Erdman strain of M. tuberculosis can cause a significant 2-fold decrease in the intracellular levels of GSH in macrophages.

**TABLE 1:** Levels of total GSH, TNF-α, and IL-10 in uninfected and M. tuberculosis-infected THP-1 cells^*a*^

Infection status and treatment	GSH concn (μmol/g protein)	TNF-α concn (pg/ml)	IL-10 concn (pg/ml)
M. tuberculosis uninfected	27.64 ± 0.413	592.1 ± 104.6	95.09 ± 10.28
M. tuberculosis infected	17 ± 0.017§§§§	4,298 ± 392.4§§§	75.22 ± 22.58
M. tuberculosis infected plus treatment with:			
NAC	32.07 ± 3.663*	379.2 ± 91.1###	16.52 ± 7.057
INH	26.15 ± 1.114##	2,830 ± 787.3	48.82 ± 19.69
INH + NAC	27.13 ± 2.815#	144.9 ± 26.8*, ###	12.88 ± 6.316
RIF	20.27 ± 0.040***, ####	2,409 ± 312.8##	59.12 ± 20.93
RIF + NAC	49.02 ± 7.837*, #	170 ± 83.99***, ####	29.12 ± 12.08
EMB	47.92 ± 13.73	2,604 ± 587.8#	94.7 ± 29.63
EMB + NAC	70.36 ± 9.504#	26.9 ± 1.302**, ####	27.48 ± 15.53
PZA	24.02 ± 0.032***, ####	2,333 ± 247.2##	59.44 ± 26.53
PZA + NAC	29.02 ± 2.536#	202.5 ± 50.27****, ###	19.76 ± 9.198

a The GSH measurements are the mean plus or minus the standard error for the uninfected control group and each infection category. The TNF-α and IL-10 cytokine measurements are the mean plus or minus the standard error for the uninfected control group and each of the infection categories. *, *P* < 0.05 when the group treated with antibiotic plus NAC is compared to the group treated with antibiotic alone or the M. tuberculosis-infected group treated with NAC is compared to the infected control group; **, *P* < 0.005 when the group treated with antibiotic plus NAC is compared to the group treated with antibiotic alone or the infected group treated with NAC is compared to the infected control group; ***, *P* < 0.0005 when the group treated with antibiotic plus NAC is compared to the group treated with antibiotic alone or the infected group treated with NAC is compared to the infected control group; ****, *P* < 0.00005 when the group treated with antibiotic plus NAC is compared to the group treated with antibiotic alone or the infected group treated with NAC is compared to the infected control group; #, *P* < 0.05 when the group treated with each antibiotic is compared to the infected control group; ##, *P* < 0.005 when the group treated with each antibiotic is compared to the infected control group; ###, *P* < 0.0005 when the group treated with each antibiotic is compared to the infected control group; ####, *P* < 0.00005 when the group treated with each antibiotic is compared to the infected control group; §§§, *P* < 0.0005 when the infected control group is compared to the uninfected control group; §§§§, *P* < 0.00005 when the infected control group is compared to uninfected control group.

### Measurement of GSH levels, bacterial survival, TNF-α and IL-10 levels, and ROS production in M. tuberculosis-infected and M. tuberculosis-infected plus NAC-treated THP-1 cells.

**FIG 2:**
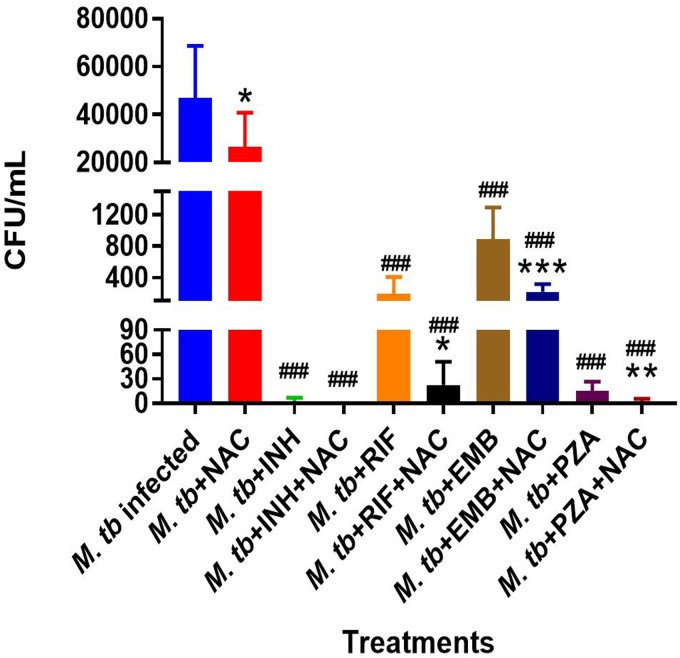
Survival of M. tuberculosis inside macrophages treated with first-line antibiotics in the presence and absence of NAC. THP-1 cells were cultured in a medium of RPMI and 10% FBS and allowed to differentiate into macrophages by addition of PMA at a concentration of 10 ng/ml. There was a significant decrease in bacterial numbers when M. tuberculosis-infected macrophages were treated with NAC. INH plus NAC treatment resulted in the clearance of M. tuberculosis infection, whereas treatment with INH only did not. There was a significant reduction in the bacterial numbers when THP-1 cells were treated with RIF plus NAC compared to the numbers when they were treated with RIF only. We also observed a significant reduction in the bacterial numbers when THP-1 cells were treated with EMB plus NAC compared to the numbers when they were treated with EMB only. Treatment of M. tuberculosis-infected macrophages with PZA plus NAC resulted in a significant reduction in bacterial numbers compared to those obtained with treatment with PZA only. Data represent the means ± SE from 6 trials. *, *P* < 0.05 when comparing the antibiotic plus NAC to the antibiotic alone or infected NAC to infected control. **, *P* < 0.005 when comparing the antibiotic plus NAC to the antibiotic alone or infected NAC to infected control. ***, *P* < 0.0005 when comparing the antibiotic plus NAC to the antibiotic alone or infected NAC to infected control. ###, *P* < 0.0005 when comparing each antibiotic category to the infected control.

**FIG 3:**
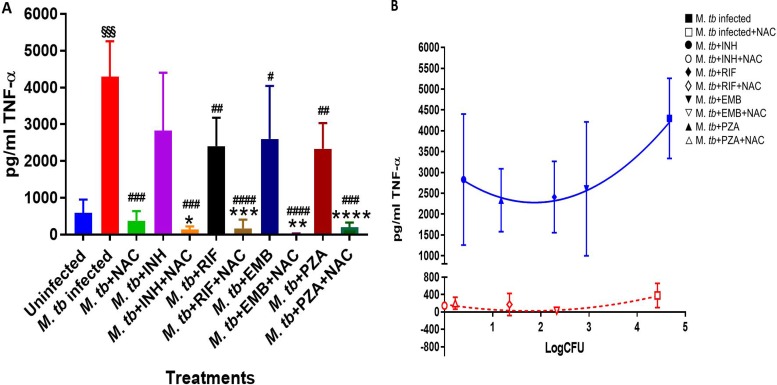
TNF-α levels in M. tuberculosis-infected macrophages treated with first-line antibiotics in the presence and absence of NAC. The assay of TNF-α was performed using an ELISA Ready-Set-Go kit from eBioscience. There was a significant increase in the levels of TNF-α when macrophages were infected with M. tuberculosis. There was a significant decrease in the levels of TNF-α when macrophages were infected with M. tuberculosis and treated with NAC. Additionally, there was a significant decrease in the levels of TNF-α when M. tuberculosis-infected macrophages were treated with INH, INH plus NAC, RIF, RIF plus NAC, EMB, EMB plus NAC, and PZA plus NAC. Data represent the means ± SE from 6 trials. *, *P* < 0.05 when comparing the antibiotic plus NAC to the antibiotic alone or infected NAC to infected control. **, *P* < 0.005 when comparing the antibiotic plus NAC to the antibiotic alone or infected NAC to infected control. ***, *P* < 0.0005 when comparing the antibiotic plus NAC to antibiotic alone or infected NAC to infected control. ****, *P* < 0.00005 when comparing the antibiotic plus NAC to antibiotic alone or infected NAC to infected control. #, *P* < 0.05 when comparing each antibiotic category to the infected control. ##, *P* < 0.005 when comparing each antibiotic category to the infected control. ###, *P* < 0.0005 when comparing each antibiotic category to the infected control. ####, *P* < 0.00005 when comparing each antibiotic category to the infected control. §§§, *P* < 0.0005 when comparing infected control to uninfected control. (B) Correlation between TNF-α production and M. tuberculosis survival inside untreated, NAC-treated, INH-treated, INH- and NAC-treated, RIF-treated, RIF- and NAC-treated, EMB-treated, EMB- and NAC-treated, PZA-treated, and PZA- and NAC-treated macrophages.

**FIG 4:**
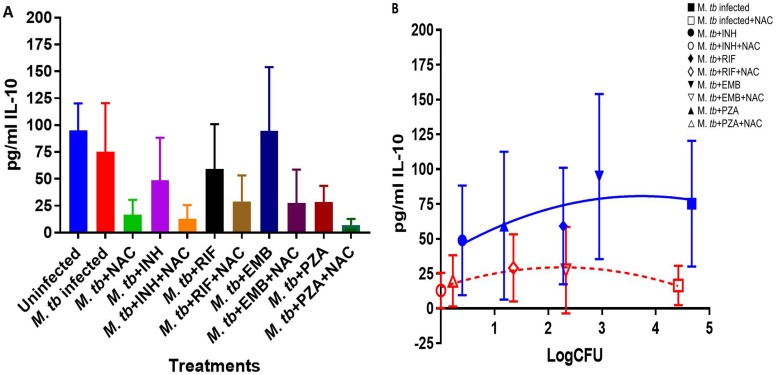
IL-10 levels in M. tuberculosis-infected macrophages treated with first-line antibiotics in the presence and absence of NAC. IL-10 was measured using an ELISA Ready-Set-Go kit from eBioscience. Although there was a decrease in the levels of IL-10 when M. tuberculosis-infected macrophages were treated with NAC, INH, INH plus NAC, RIF, RIF plus NAC, and EMB plus NAC, this difference was not found to be statistically significant. There was a significant decrease in the levels of IL-10 when macrophages were infected with M. tuberculosis and treated with PZA plus NAC and a slight decrease when samples were treated with PZA (not significant). Data represent the means ± SE from 6 trials. (B) Correlation between IL-10 production and M. tuberculosis survival inside untreated, NAC-treated, INH-treated, INH- and NAC-treated, RIF-treated, RIF- and NAC-treated, EMB-treated, EMB- and NAC-treated, PZA-treated, and PZA- and NAC-treated macrophages.

**FIG 5:**
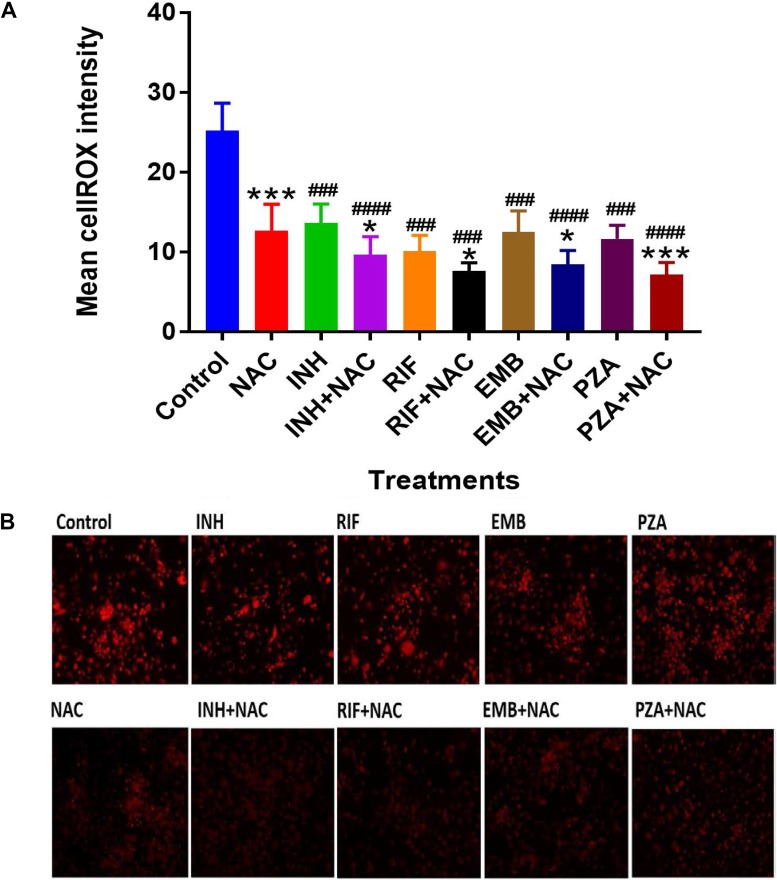
Levels of ROS in M. tuberculosis-infected macrophages treated with first-line antibiotics in the presence and absence of NAC. THP-1 cells were fixed by 4% paraformaldehyde, and ROS measurements were performed by fluorescent staining using a cellROX Red assay kit from Life Technologies. There was a significant decrease in ROS amount in M. tuberculosis-infected samples treated with NAC. A significant decrease of ROS levels was also observed when M. tuberculosis-infected samples were treated with INH, INH plus NAC, RIF, RIF plus NAC, EMB, EMB plus NAC, PZA, and PZA plus NAC. There was also a significant decrease in ROS levels in antibiotic-plus-NAC categories compared to stand-alone antibiotic categories. The mean cell intensity was analyzed by ImageJ software. Data represent means ± SE from 6 trials. *, *P* < 0.05 when comparing the antibiotic plus NAC to the antibiotic alone or infected NAC to infected control. ***, *P* < 0.0005 when comparing the antibiotic plus NAC to the antibiotic alone or infected NAC to infected control. ###, *P* < 0.0005 when comparing each antibiotic category to the infected control. ####, *P* < 0.00005 when comparing each antibiotic category to the infected control.

**FIG 6:**
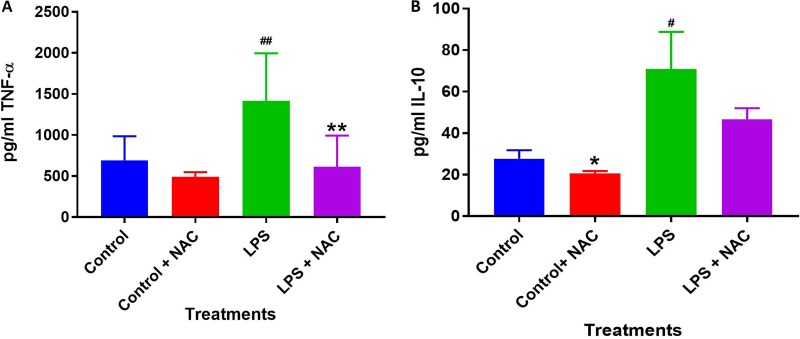
The direct effects of NAC on TNF-α and IL-10 levels of untreated and LPS-treated THP-1 macrophages. A reduction of TNF-α and IL-10 levels was observed with NAC treatment for both untreated and LPS-treated THP-1 macrophages. LPS-treated THP-1 macrophages had a significant increase in both TNF-α and IL-10 levels compared to the untreated group. Data represent means ± SE from 6 trials. *, *P* < 0.05, indicates significant decrease in TNF-α and IL-10 levels with NAC treatment. **, *P* < 0.005, indicates significant decrease in TNF-α and IL-10 levels with NAC treatment. #, *P* < 0.05, indicates significant increase in TNF-α and IL-10 levels with LPS treatment. ##, *P* < 0.005, indicates significant increase in TNF-α and IL-10 levels with LPS treatment.

 Treatment of M. tuberculosis-infected macrophages with NAC resulted in a significant 2-fold increase in the intracellular levels of GSH compared to those in the M. tuberculosis-infected sham-treated control group (Fig. 1; Table 1). NAC treatment therefore restored the levels of GSH in M. tuberculosis-infected macrophages. The restoration of the levels of GSH was significantly interconnected with the reduction of intracellular survival of M. tuberculosis inside the NAC-treated macrophages (Fig. 2; Table 2). There was a statistically significant 6-fold increase in the levels of TNF-α in M. tuberculosis-infected macrophages compared to those in the uninfected control group (Fig. 3A and B; Table 1). Our results imply that the excess production of TNF-α by M. tuberculosis-infected macrophages is correlated with the decrease in the amount of GSH measured and thereby favors the intracellular survival of M. tuberculosis. GSH replenishment of M. tuberculosis-infected macrophages resulted in a significant 8-fold decrease in the levels of TNF-α compared to those in the M. tuberculosis-infected control group (Fig. 3A and B; Table 1). Importantly, NAC treatment of M. tuberculosis-infected macrophages resulted in a 4-fold decrease in the levels of IL-10 compared to those in the M. tuberculosis-infected sham-treated control group (Fig. 4A and B; Table 1). The addition of NAC to lipopolysaccharide (LPS)-treated uninfected macrophages likewise resulted in a significant downregulation of TNF-α production, as well as a substantial decrease in the levels of IL-10 (Fig. 5A and B). A profound red fluorescence was observed for the control category after staining with the CellROX Deep Red reagent. However, after NAC treatment a significant reduction in the mean fluorescent intensity was detected, indicative of diminished levels of reactive oxygen species (ROS) (Fig. 6A and B). These results further assert that restoring redox homeostasis and cytokine balance should likewise improve the control of intracellular M. tuberculosis infection.

**TABLE 2:** Survival of Erdman strain of M. tuberculosis inside THP-1 macrophages^*a*^

Infection status and treatment	No. of CFU/ml
M. tuberculosis infected	46,884 ± 7,255
M. tuberculosis infected plus treatment with:	
NAC	26,520 ± 4,496*
INH	2.5 ± 1.306###
INH + NAC	0 ± 0###
RIF	194.2 ± 61.86###
RIF + NAC	22.5 ± 8.269*, ###
EMB	888.3 ± 117.2###
EMB + NAC	215.8 ± 28.75***, ###
PZA	15 ± 3.371###
PZA + NAC	1.667 ± 1.124**, ###

a The data represent the mean number of CFU per milliliter plus or minus the standard error for each infection category. *, *P* < 0.05 when the group treated with antibiotic plus NAC is compared to the group treated with antibiotic alone or the infected group treated with NAC is compared to the infected control group; **, *P* < 0.005 when the group treated with antibiotic plus NAC is compared to the group treated with antibiotic alone or the infected group treated with NAC is compared to the infected control group; ***, *P* < 0.0005 when the group treated with antibiotic plus NAC is compared to the group treated with antibiotic alone or the infected group treated with NAC is compared to the infected control group; #, *P* < 0.05 when the group treated with each antibiotic is compared to the infected control group; ##, *P* < 0.005 when the group treated with each antibiotic is compared to the infected control group; ###, *P* < 0.0005 when the group treated with each antibiotic is compared to the infected control group.

### Quantification of GSH levels, M. tuberculosis survival, TNF-α and IL-10 levels, and ROS production in M. tuberculosis-infected, M. tuberculosis-infected and INH-treated, and M. tuberculosis-infected and INH-NAC-treated THP-1 cells.

There was a significant increase in the levels of GSH in M. tuberculosis-infected macrophages treated with INH and INH-NAC compared to those in the infected sham-treated controls (Fig. 1; Table 1). Treatment with INH-NAC resulted in the complete clearance of M. tuberculosis infection, whereas treatment with INH alone did not (Fig. 2; Table 2). Treatment with either INH or the combination of INH and NAC resulted in almost a 10,000-fold decrease in the viability of M. tuberculosis compared to that with treatment with NAC only (Fig. 2; Table 2) and a roughly 15,000-fold decrease in the viability of M. tuberculosis compared to that in the sham-treated control group (Fig. 2; Table 2). These results further emphasize that a reduction in the M. tuberculosis burden can restore the intracellular levels of GSH. Treatment of M. tuberculosis-infected macrophages with INH resulted in a statistically significant decrease in the levels of TNF-α compared to those in the infected sham-treated control group. Treatment of M. tuberculosis-infected macrophages with INH-NAC also resulted in a statistically significant decrease in the levels of TNF-α (Fig. 3A and B; Table 1). INH-NAC treatment resulted in a further reduction in the levels of TNF-α in comparison to those obtained by treatment with INH alone. Consistent with our findings from the TNF-α assay, INH treatment of M. tuberculosis-infected macrophages also resulted in a reduction in the levels of IL-10 compared to those in the infected sham-treated controls. INH-NAC treatment caused an additional decrease in the levels of IL-10 (Fig. 4A and B; Table 1). These findings specify the effects of NAC in diminishing the levels of TNF-α and IL-10. An intense red fluorescence was observed for the INH treatment group after CellROX Deep Red staining. However, after NAC treatment was simultaneously administered, a significant reduction in the mean intensity was observed (Fig. 6A and B).

### Quantification of GSH levels, M. tuberculosis survival, TNF-α and IL-10 levels, and ROS production in M. tuberculosis-infected, M. tuberculosis-infected and RIF-treated, and M. tuberculosis-infected and RIF-NAC-treated THP-1 cells.

RIF treatment of infected macrophages resulted in a significant increase in GSH levels compared to those in the infected sham-treated control group. Importantly, RIF-NAC treatment resulted in further enhancement in the levels of GSH and a statistically significant increase compared both to those in the infected sham-treated control group and also to those in the group treated with RIF alone (Fig. 1; Table 1).

The notable increase in the levels of GSH in the RIF-NAC treatment group was accompanied by a significant 8-fold reduction in the intracellular viability of M. tuberculosis compared to that with treatment with RIF alone (Fig. 2; Table 2). When comparing the efficacy of INH and RIF, there were also approximately 70 times more intracellular M. tuberculosis bacteria when macrophages were treated with RIF than when they were treated with INH and 25 times more bacteria when macrophages were treated with RIF with NAC than when they were treated with INH with NAC (Fig. 2; Table 2). RIF treatment reduced the bacterial burden by almost 200 times compared to sham treatment (Fig. 2; Table 2), and treatment with RIF-NAC reduced the M. tuberculosis counts by 1,600 times. Treatment of M. tuberculosis-infected macrophages with the combination of RIF and NAC also resulted in a statistically significant decrease in the levels of TNF-α compared to those in both the infected sham-treated control group and the group treated with RIF alone (Fig. 3A and B; Table 1). Treatment with RIF caused a 50% reduction in the levels of TNF-α compared to those in the sham-treated controls (Fig. 3A and B; Table 1). Treatment with RIF-NAC resulted in an 80-fold reduction in the levels of TNF-α in comparison to those in the sham-treated controls (Fig. 3A and B; Table 1). RIF administered alone was not able to lower the levels of TNF-α as much as INH by itself, but the addition of NAC to RIF lowered the TNF-α levels 8 times more than INH and NAC treatment (Fig. 3A and B; Table 1). RIF treatment resulted in a decrease in the levels of IL-10 compared to those in the sham-treated control group (Fig. 4A and B; Table 1). Treatment of M. tuberculosis-infected macrophages with the combination of RIF and NAC resulted in a further decrease in the levels of IL-10 compared to those in the control group and the group treated with RIF, but neither of these decreases was statistically significant (Fig. 4A and B; Table 1). The trends for IL-10 were very similar when RIF and INH treatments were compared. However, INH was twice as effective in lowering the levels of IL-10 in macrophage cultures (Fig. 4A and B; Table 1). An intense red fluorescence was also observed for the RIF-treated group after CellROX Deep Red staining, and again after NAC treatment was simultaneously administered, a significant reduction in the mean intensity was observed (Fig. 6A and B).

### Quantification of GSH levels, M. tuberculosis survival, TNF-α and IL-10 levels, and ROS production in M. tuberculosis-infected, M. tuberculosis-infected and EMB-treated, and M. tuberculosis-infected and EMB-NAC-treated THP-1 cells.

GSH levels were increased by 2-fold in the macrophages treated with EMB compared to those in the infected sham-treated control group, although this increase was not significant. However, when macrophages were treated with EMB-NAC, there was a statistically significant 3-fold increase in the levels of GSH compared to those in the sham-treated control group (Fig. 1; Table 1). Consistent with the increase in the levels of GSH, there was 4-fold decrease in the intracellular survival of M. tuberculosis inside the EMB-NAC-treated macrophages compared to that of M. tuberculosis inside macrophages treated with EMB alone (Fig. 2; Table 2). The infected macrophages treated with EMB alone had significantly reduced levels of TNF-α compared to those in the infected sham-treated control group (Fig. 3A and B; Table 1). EMB-NAC treatment resulted in a further decrease in the levels of TNF-α in comparison to those in the sham-treated control and the group treated with EMB alone. Treatment with EMB alone and EMB plus NAC resulted in more than 50% and 80% decreases in the levels of TNF-α, respectively, in comparison to those in the sham-treated control group (Fig. 3A and B; Table 1). Although the difference was not significant, a distinct reduction in the levels of IL-10 was observed when M. tuberculosis-infected macrophages were treated with EMB plus NAC (Fig. 4A and B; Table 1). Similarly, the macrophages in the EMB antibiotic treatment category likewise displayed an intense red fluorescence after CellROX Deep Red staining, and after NAC cotreatment, a significant reduction in the mean intensity was once again observed (Fig. 6A and B).

### Quantification of GSH levels, M. tuberculosis survival, TNF-α and IL-10 levels, and ROS production in M. tuberculosis-infected, M. tuberculosis-infected and PZA-treated, and M. tuberculosis-infected and PZA-NAC-treated THP-1 cells.

The levels of GSH in PZA-treated macrophages were significantly elevated compared to those in the infected sham-treated control group (Fig. 1; Table 1). There was a further enhancement in the levels of GSH when M. tuberculosis-infected macrophages were treated with PZA plus NAC (Fig. 1; Table 1). Treatment with the combination of PZA and NAC led to a statistically significant decrease in the number of CFU of M. tuberculosis compared to that in the group treated with PZA alone (Fig. 2; Table 2). PZA was more effective in lowering the bacterial load than both RIF and EMB (Fig. 2; Table 2). Additionally, the treatment with PZA given in conjunction with NAC was as effective as the treatment with INH alone (Fig. 2; Table 2). The combined treatment with PZA and NAC produced a statistically significant decrease in the levels of TNF-α compared to those in both the infected sham-treated control group and the group treated with PZA alone (Fig. 3A and B; Table 1). The levels of TNF-α after treatment with PZA were similar to those in the groups treated with EMB and RIF alone (Fig. 3A and B; Table 1). IL-10 levels were decreased in M. tuberculosis-infected macrophages treated with PZA and PZA plus NAC in comparison to those in the sham-treated control group (Fig. 4A and B; Table 1). Although the difference was not statistically significant, the PZA-treated macrophages showed a marked decrease in IL-10 levels compared to those in the sham-treated control group. Importantly, treatment with PZA plus NAC resulted in a statistically significant decrease in the levels IL-10 compared to those in the sham-treated control group (Fig. 4A and B; Table 1). Furthermore, of all the first-line antibiotics tested, PZA treatment resulted in the lowest levels of IL-10 measured. Similar to the findings for the aforementioned antibiotics, macrophages treated with PZA alone also displayed an intense red fluorescence after CellROX Deep Red staining, and consistent with the findings for the previous antibiotic categories, after NAC cotreatment a significant reduction in the mean intensity was detected (Fig. 6A and B).

## DISCUSSION

Immunocompromised individuals, such as HIV-positive subjects, are increasingly susceptible to M. tuberculosis infection. Additionally, the rapid increase in the number of people living with drug-resistant TB (DR-TB) and TB and HIV coinfection generates additional challenges for global targets of TB elimination. We previously reported that the virulent laboratory strain of M. tuberculosis H37Rv is sensitive to physiological concentrations of GSH (5 mM) when grown i*n vitro* (30, 31). We also found that enhancing the levels of GSH in human macrophages by treatment with NAC (10 or 20 mM) resulted in inhibition in the growth of intracellular H37Rv (30–34). Thus, GSH has direct antimycobacterial activity, functioning as an effector molecule in the innate defense against M. tuberculosis infection (30–36). These results reveal a novel and potentially important innate defense mechanism adopted by human macrophages to control M. tuberculosis infection (30–36). We also reported that GSH in combination with cytokines, such as IL-2 and IL-12, enhances the functional activity of NK cells to inhibit the growth of M. tuberculosis inside human monocytes (35). We then demonstrated that GSH activates the functions of T lymphocytes to control M. tuberculosis infection inside human monocytes (36). These results indicate that GSH inhibits the growth of M. tuberculosis both by direct antimycobacterial effects and by enhancing the functions of immune cells (30–33, 35). Finally, we demonstrated that the levels of GSH are significantly compromised in individuals with active pulmonary TB (34).

Therefore, we tested the synergistic effects of NAC (as a GSH precursor) and suboptimal levels of individual first-line anti-TB drugs (INH, RIF, EMB, and PZA) in mediating the control of M. tuberculosis infection inside THP-1 macrophages. The THP-1 cell line is an immortalized monocyte cell line derived from the blood of a childhood case of acute monocytic leukemia (37, 38). Each antibiotic was administered only once throughout the trial at its MIC. Each antibiotic was administered at its MIC to ensure that complete bacterial clearance was not achieved by supplementation of the antibiotics alone.

Our results demonstrate that macrophages treated with suboptimal levels of each of the first-line antibiotics in conjunction with NAC resulted in a significant reduction in the intracellular survival of M. tuberculosis compared to that after the administration of suboptimal levels of unaccompanied antibiotics (Fig. 2; Table 2). In fact, complete clearance was observed for the INH-treated group after NAC was added (Fig. 2; Table 2). These novel findings illustrate the synergistic effects of NAC/GSH and antibiotics in improving the macrophage's ability to control intracellular M. tuberculosis and suggest that GSH has suitable potential as an adjunct with the aforementioned first-line antibiotics in clearing an M. tuberculosis infection and aiding in the cessation of drug-resistant strains of M. tuberculosis.

We observed a significant decrease in the levels of intracellular GSH in M. tuberculosis-infected macrophages compared to those in uninfected macrophages (Fig. 1A; Table 1). These results indicate that M. tuberculosis infection can cause intracellular GSH depletion, which in turn can promote M. tuberculosis survival and replication inside the host cells (Fig. 1 and 2; Tables 1 and 2). Furthermore, enhancing the levels of GSH in M. tuberculosis-infected macrophages by treatment with NAC (Fig. 1 and Table 1) resulted in a significant reduction in the intracellular survival of M. tuberculosis (Fig. 2 and Table 2). Treatment of M. tuberculosis-infected macrophages with each of the first-line antibiotics resulted in restoration in the levels of GSH and a statistically significant increase (except with EMB treatment [*P* < 0.05]) compared to those in the M. tuberculosis-infected sham-treated control group (Fig. 1 and Table 1). These results indicate that the use of antibiotics to limit the M. tuberculosis burden in macrophages enabled the host cells to restore the levels of GSH and improved their ability to combat the infection. Importantly, treatment of M. tuberculosis-infected macrophages with NAC in conjunction with each of the first-line antibiotics resulted in a statistically significant notable increase in the levels of GSH in all the treated groups (Fig. 1 and Table 1). The levels of GSH detected in macrophages treated with a first-line antibiotic in combination with NAC were consistently higher than those in the sham-treated control macrophages and macrophages treated with antibiotic alone (Fig. 1 and Table 1). Antibiotic treatment given in conjunction with NAC resulted in the decreased production of TNF-α and IL-10 by the macrophages. In the context of an M. tuberculosis infection, TNF-α is an inflammatory cytokine produced by macrophages and is particularly important in promoting the formation and maintenance of a granuloma, whereas IL-10 is an immunosuppressive cytokine which acts as a negative regulator of the immune response associated with fighting the infection (39–41). TNF-α is a diverse cytokine which has been shown to functionally aid in the formation and maintenance of a granuloma as well as to play a critical role in the host defense against M. tuberculosis in both the acute phase and the chronic phase of infection (42–44). However, at high levels, TNF-α is also implicated as the source of many inflammatory and autoimmune diseases and has been shown to cause severe tissue damage when it is overexpressed during an M. tuberculosis infection (45, 46). When the combination of NAC and each of the first-line antibiotics was administered, the TNF-α levels in each group were significantly reduced from the extremely high levels seen in the infected sham-treated control group and presented closer to those in the uninfected group (Fig. 3A and B and Table 1). Our results indicate that NAC treatment can modulate the levels of TNF-α in a manner that it is significant enough to maintain a healthy granuloma but cannot cause cellular damage. Increased levels of the cytokine IL-10 can dampen the effector responses against an M. tuberculosis infection by inhibiting phagosome-lysosome fusion within macrophages (46–48). Although the difference was not statistically significant, a notable reduction in the levels of IL-10 was observed for every antibiotic treatment group when the antibiotic was administered with NAC (Fig. 4A and B and Table 1). Additionally, when NAC was supplemented unaided, a reduction of roughly double the magnitude of IL-10 was likewise detected (Fig. 4A and B and Table 1). These data imply that NAC supplementation supports the immune response in favor of eliminating an M. tuberculosis infection by reducing the levels of IL-10, allowing macrophage phagosome maturation and, thus, enhanced pathogen elimination. Our findings illustrate that, in addition to the direct antimycobacterial effects, the GSH enhancement improved the ability of the first-line antibiotics to limit intracellular M. tuberculosis infection and modulated cytokine production by macrophages.

The robust red fluorescence observed for the control group and the groups treated with each of the antibiotics alone after CellROX Deep Red staining was mitigated after NAC treatment/cotreatment. This significant reduction in the mean intensity signifies attenuation of the extent of ROS production (Fig. 6A and B). Diminished ROS production implies that the extent of overall oxidative stress decreased as well. These results further suggest that restoring redox homeostasis correlates with improved immune functionality and control over an M. tuberculosis infection.

When all four antibiotics were administered together at their MICs, complete mycobacterial clearance was observed with and without the addition of NAC (data not shown). A trend similar to that seen with the supplementation of individual antibiotics was observed, where a significant increase in the levels of GSH was detected from the administration of all four antibiotics without NAC and a further increase was detected once NAC was additionally added (data not shown). Likewise, a reduction in the levels of TNF-α and IL-10 was observed with the administration of all four antibiotics without NAC supplementation, and the reduction was statistically significant once NAC was also administered (data not shown).

Our findings highlight that GSH exhibits more physiological significance than just intracellular redox homeostasis, advocating that its enhancement aids in cytokine balance as well as augments the ability of first-line anti-TB drugs to clear an M. tuberculosis infection. Therefore, we believe that including GSH (NAC) in the antibiotic treatment of TB not only would limit cellular damage by means of redox balance and subsequently reduce the potential toxicity of the anti-TB medications but also could possibly limit the dosage required to cause complete bacterial clearance as well as help combat further emergences of DR-TB strains.

## MATERIALS AND METHODS

### THP-1 cell culture.

THP-1 cells were maintained in RPMI medium (Sigma-Aldrich) containing 10% fetal bovine serum (FBS; Sigma-Aldrich) and 2 mM glutamine (Sigma-Aldrich). For the assays, the cell suspension was centrifuged at 2,000 rpm for 15 min, and the pellet was resuspended in RPMI medium containing 10% FBS. The cell numbers in the suspension were determined by trypan blue dye exclusion staining. THP-1 cells (2 × 10^5^ cells/well) were distributed in 24-well plates (Corning), treated with phorbol 12-myristate 13-acetate (PMA) at a 10-ng/ml concentration, and incubated overnight at 37°C in 5% CO_2_ to induce the differentiation to macrophages. Following overnight incubation, the media in the wells were replaced with fresh media.

### Preparation of bacteria for infection assays.

The Erdman strain of M. tuberculosis (which was gifted by Selvakumar Subbian, Rutgers New Jersey Medical School, Biomedical and Health Sciences) was used for all our infection studies. The Erdman strain of M. tuberculosis (referred to as M. tuberculosis) has a slightly faster doubling time and is more virulent than the standard laboratory strain H37Rv (49). M. tuberculosis was cultured in 7H9 medium (Middlebrook) supplemented with albumin-dextrose complex (ADC) at 37°C until processing. M. tuberculosis was processed for infection once the static culture was at the peak logarithmic phase of growth (optical density at 600 nm, between 0.5 and 0.8) and subsequently washed and resuspended in sterile 1× phosphate-buffered saline (PBS). Bacterial clumps were dispersed by vortexing five times with 3-mm sterile glass beads at 2-min intervals. The bacterial suspension was then filtered using a 5-μm-pore-size syringe filter (Millipore) to remove any remaining bacterial aggregations. The single-cell suspension of processed M. tuberculosis was serially diluted and plated on 7H11 agar to determine the bacterial numbers in the processed stock. Aliquots of processed bacterial stocks were frozen at −80°C. At the time of infection, the processed frozen stocks of M. tuberculosis were thawed and used for infection. All infection studies and handling of the M. tuberculosis were done inside a certified biosafety level 3 (BSL-3) facility.

### THP-1 macrophage infection and treatment.

Differentiated macrophages were infected with processed M. tuberculosis at a multiplicity of infection (MOI) of 0.1:1 (bacterium-to-macrophage ratio). Infected macrophages were incubated for 1 h and then successively washed 3 times with warm 1× PBS to remove the unphagocytosed bacteria. Infected macrophages then either were sham treated or received a onetime treatment with the MIC of the respective antibiotic with or without NAC addition. NAC (10 mM) was administered at 3 equal intervals throughout the trial. The treatment concentrations administered were as follows: INH at 0.125 μg/ml, INH at 0.125 μg/ml plus NAC at 10 mM (3 times), RIF at 0.125 μg/ml, RIF at 0.125 μg/ml plus NAC at 10 mM (3 times), EMB at 8.0 μg/ml, EMB at 8.0 μg/ml plus NAC at 10 mM (3 times), PZA at 50 μg/ml, and PZA at 50 μg/ml plus NAC at 10 mM (3 times). The infected cells were maintained at 37°C in 5% CO_2_ until they were terminated at 1 h and 12 days postinfection to determine the intracellular survival of M. tuberculosis. A successive, identical experiment was performed in the absence of M. tuberculosis infection, and lipopolysaccharide (LPS) was alternatively administered (Sigma-Aldrich) at 1 μg/ml as a positive-control method to determine the direct effects of NAC in altering the production of TNF-α and IL-10.

### Termination of macrophages and CFU assay.

Termination of infected macrophages was performed by collecting and storing the cell-free supernatants and lysing THP-1 cells using 250 μl of ice-cold, sterile 1× PBS. Cell lysates collected from the wells were vigorously vortexed and then subjected to freeze-thaw cycles to ensure the complete lysis of the macrophages. The collected lysates and supernatants were then diluted in sterile 1× PBS and plated on 7H11 medium (HiMedia) enriched with ADC to evaluate M. tuberculosis survival inside the macrophages by counting the bacterial colonies.

### Quantification of GSH levels in cellular lysates.

The quantity of total glutathione present was measured by the colorimetric method using an assay kit from Arbor Assay (catalog number K006-H1). The macrophage lysates were first thoroughly mixed with an equal volume of cold 5% sulfosalicylic acid (SSA) and then incubated for 10 min at 4°C, followed by centrifugation at 14,000 rpm for 10 min. The GSH level was measured following the manufacturer's instructions. All measurements were normalized by the total protein levels, and the results are reported as the number of moles of GSH per gram of protein.

### Measurement of cytokines in culture medium.

The effects of M. tuberculosis infection, LPS administration, and antibiotic and NAC treatments in altering the production of TNF-α and IL-10 were determined by quantifying the levels of these cytokines in the macrophage supernatants collected at 12 days postinfection by enzyme-linked immunosorbent assay (ELISA) using assay kits from Affymetrix per the manufacturer's protocol.

### ROS measurements by fluorescence microscopy.

The experimental setup for each trial included the setting aside of wells for fluorescence microscopic studies. This was accomplished by allowing the cells to adhere to cover glasses positioned in the wells of tissue culture plates. The cells on the cover glasses were terminated at 12 days postinfection by fixation with 4% paraformaldehyde (PFA) for 1 h at room temperature and then washed with 1× PBS for 5 min to remove any cell debris. Reactive oxygen species (ROS) production was determined by CellROX Deep Red staining. Untreated, NAC-treated, antibiotic-treated, and antibiotic- and NAC-treated cells were treated with 5 μM the CellROX Deep Red reagent (catalog number C10422; Life Technologies) and incubated at room temperature for 30 min in the dark. The stained slides were observed under an inverted fluorescence microscope to evaluate the extent of ROS production. ImageJ software was used to quantify the mean intensity observed from the fluorescent imagery.

### Statistical analysis.

Statistical data analysis was performed using GraphPad Prism software (version 7). The levels of cytokines, GSH, and malondialdehyde and the number of CFU were compared between the untreated control group, the antibiotic-treated groups, and the groups treated with antibiotics in conjunction with NAC using the unpaired *t* test with the Welch correction. The reported values are means. A *P* value of <0.05 was considered significant.
